# Supplementary material for: ATP and nucleic acids competitively modulate LLPS of the SARS-CoV2 nucleocapsid protein
Source: Commun Biol. 2023 Jan 21;6:80. doi: 10.1038/s42003-023-04480-3 (PMC9862227; doi:10.1038/s42003-023-04480-3)
Supplement: Supplementary file 2 — SupplementalMaterials [file 42003_2023_4480_MOESM2_ESM.pdf]

ATP and nucleic acids competitively modulate LLPS of the SARS-CoV2 nucleocapsid protein

Mei Dang, Tongyang Li, Jianxing Song*
Department of Biological Sciences, Faculty of Science; National University of Singapore; 10 Kent Ridge Crescent, Singapore 119260

Supplementary Figures and Table

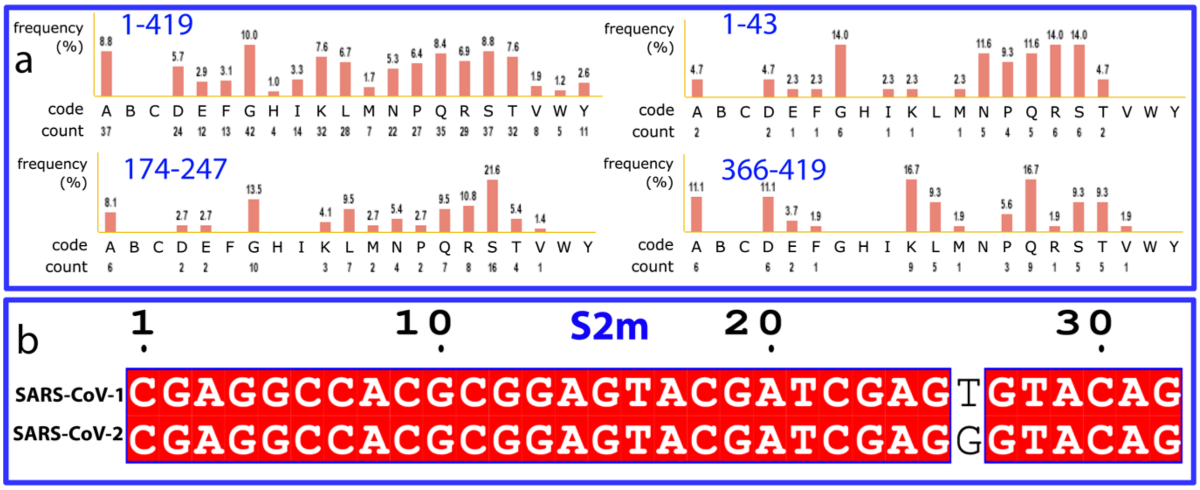

Supplementary Figure 1. Amino acid compositions of N protein and three IDRs (a); and Sequences of 32-mer S2m ssDNA of SARS-Cov-1 and SARS-CoV-2 (b).

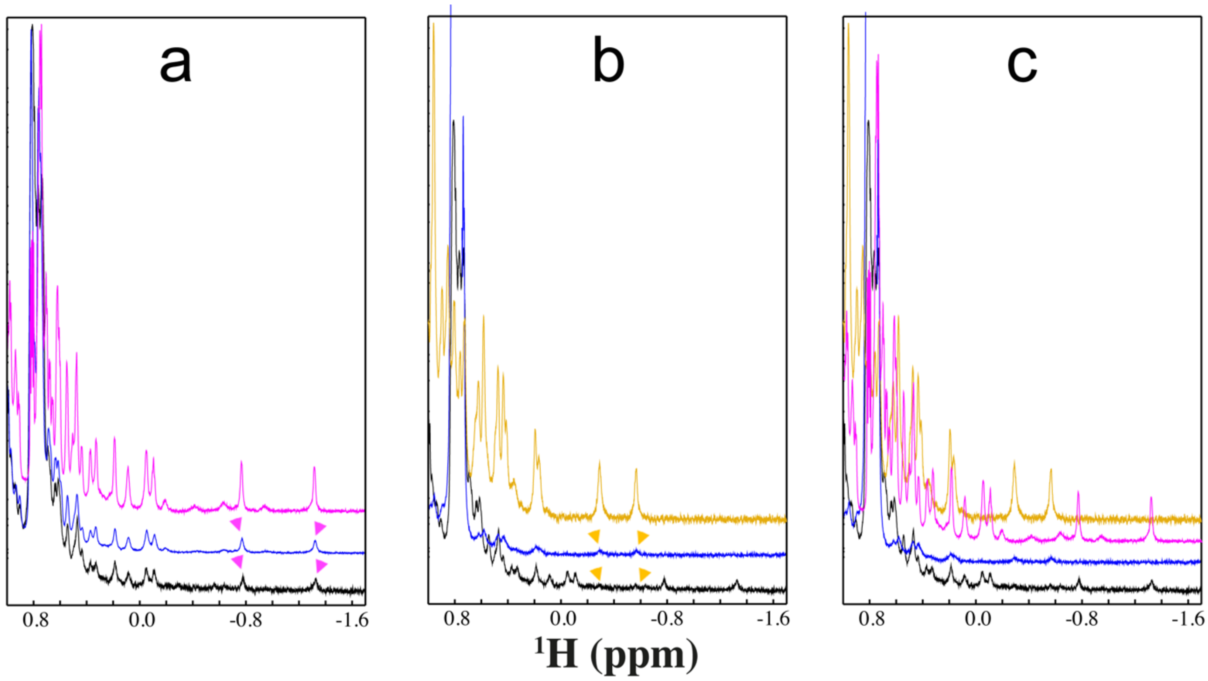

Supplementary Figure 2. NMR characterization of differentially-dissected domains of N protein.

(a) One dimensional NMR proton spectra of the side-chain regions of the full-length N protein (black), N (1-249) (blue) and NTD (purple). (b) One dimensional NMR proton spectra of the side-chain regions of the full-length N protein (black), N (175-419) (blue) and CTD (light brown). Arrows are used to indicate very up-field signature NMR signals of methyl groups of the well-folded NTD (purple) and CTD (light brown) in 1D NMR spectra of the full-length N protein, or N (1-249) or N (175-419). (c) One dimensional NMR proton spectra of the side-chain regions of the full-length N protein (black), N (175-419) (blue) and CTD (light brown), as well as NTD (purple).

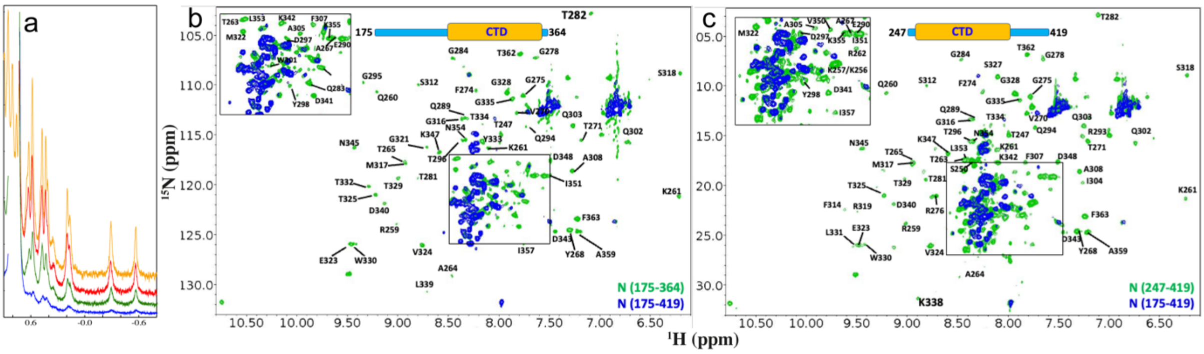

Supplementary Figure 3. NMR characterization of CTD-containing domains of N protein. (a) One dimensional NMR proton spectra of the side-chain regions of N (175-419) (blue), N (175-364) (green), N (247-419) (red) and CTD (brown). Superimposition of HSQC spectra of N (175-419) (blue) and N (175-364) (green) (b) as well as N (175-419) (blue) and (247-419) (green) (c).

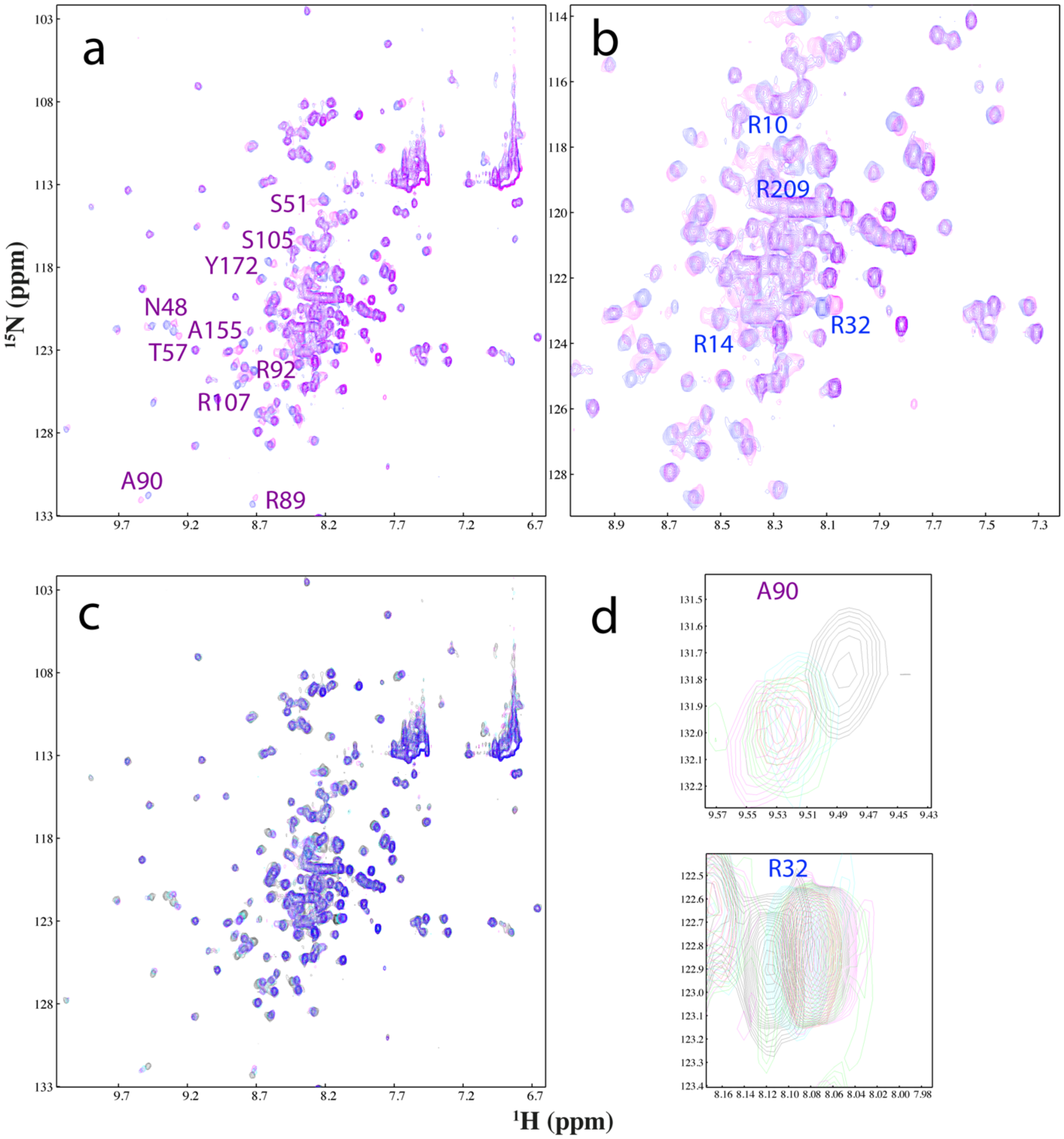

Supplementary Figure 4. ATP specifically binds residues of N (1-249).
(a) Superimposition of HSQC spectra of N (1-249) in the free state (blue) and in the presence of ATP at 10 mM (purple). (b) Zoom of the HSQC spectra showing peaks of IDR residues. (c) Superimposition of HSQC spectra of N (1-249) in the presence of ATP at different concentrations. For clarity, only spectra at four ATP concentrations are included: free state (black), ATP at 2 mM (cyan), 6 mM (purple) and 10 mM (blue). (d) Tracings of chemical shift changes of two selected residues Ala90 in NTD and Arg32 in IDR1 at different ATP concentrations: 0 mM (black), 2 mM (cyan), 4 mM (red), 6 mM (green) and 10 mM (purple).

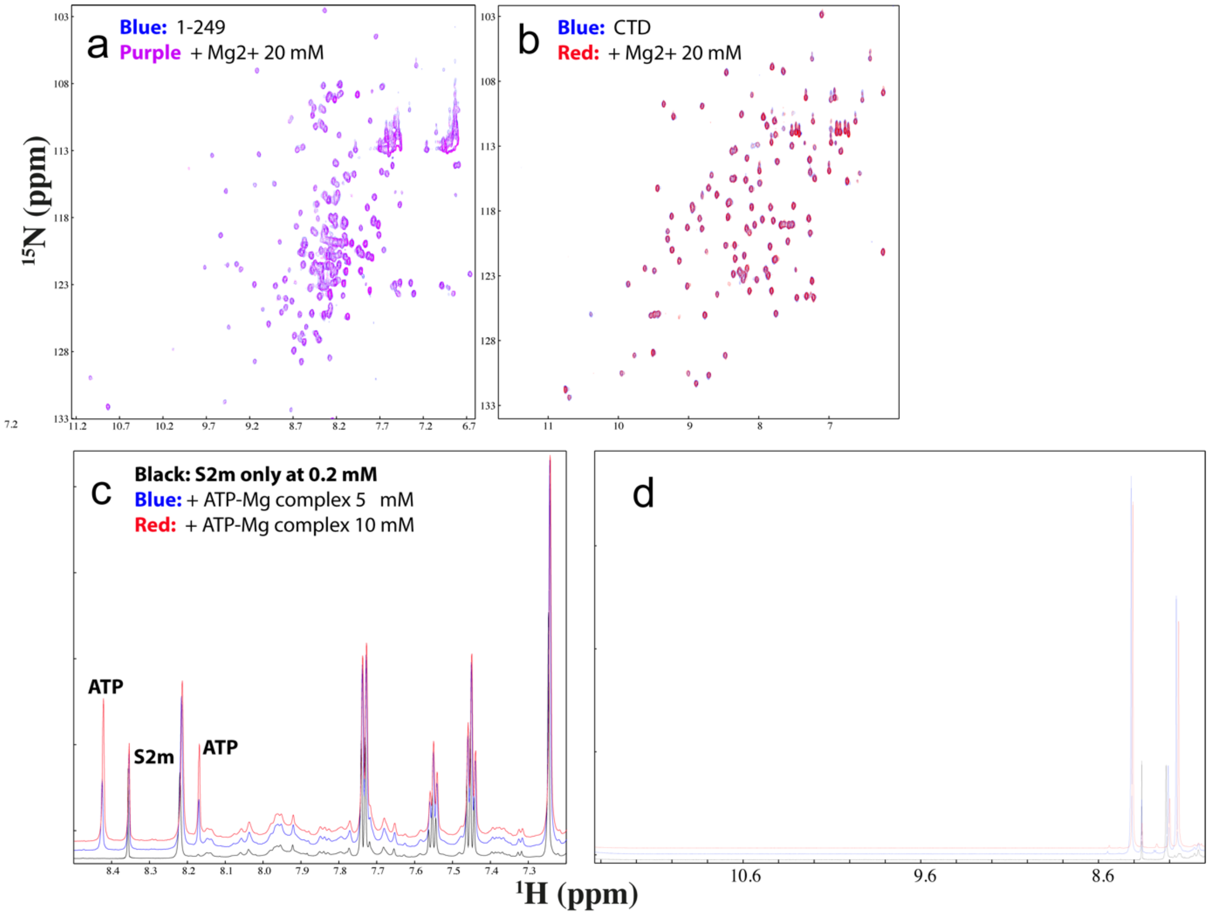

Supplementary Figure 5. No detectable interactions between Mg^{2+} and protein fragments as well as no alteration of S2m conformation by ATP-Mg^{2+} complex.

(a) Superimposition of HSQC spectra of N (1-249) in the free state (blue) and in the presence of $MgCl_2$ at 20 mM (purple). (b) Superimposition of HSQC spectra of CTD in the free state (blue) and in the presence of $MgCl_2$ at 20 mM (red). (c)-(d) 1D proton NMR spectra of the base aromatic protons and NH2 of S2m at 200 μM without ATP-**Mg^{2+}** complex (black) and with addition of ATP-**Mg^{2+}** complex at 5 mM (blue) and 10 mM (red).

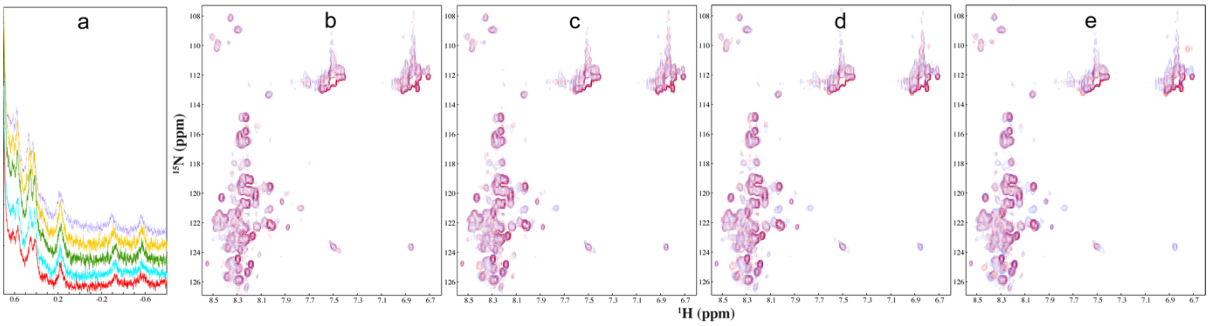

Supplementary Figure 6. ATP binds CTD of N (175-419).
(a) One dimensional NMR proton spectra of the side-chain regions of N (175-419) in the absence (blue) and in the presence of ATP at 1 mM (yellow), 4 mM (green), 6 mM (cyan) and 10 mM (red). Superimposition of HSQC spectra of N (175-419) in the absence (blue) and in the presence of ATP (red) at 1 mM (b), 4 mM (c), 6 mM (d) and 10 mM (e).

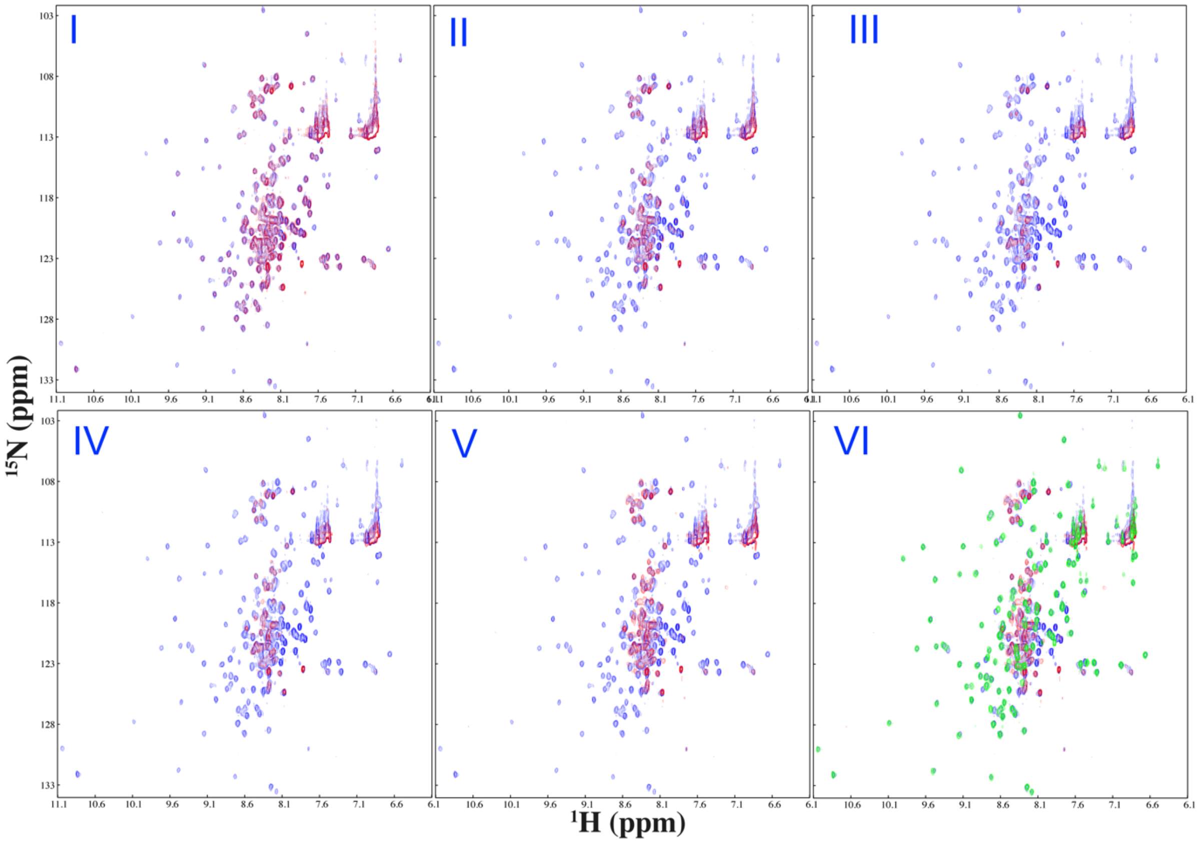

Supplementary Figure 7. NMR view of the biphasic modulation of LLPS of N (1-249) by S2m.

Superimposition of HSQC spectra of N (1-249) in the free state (blue) and in the presence of S2m (red) at 1:0.05 (I), 1:0.25 (II) and 1:0.75 (III), 1:1 (IV) and 1:2.5 (V) (1-249:S2m). (VI) Superimposition of HSQC spectra of N (1-249) in the free state (blue) and in the presence of S2m at 1:2.5 (red) as well as NTD in the free state (green).

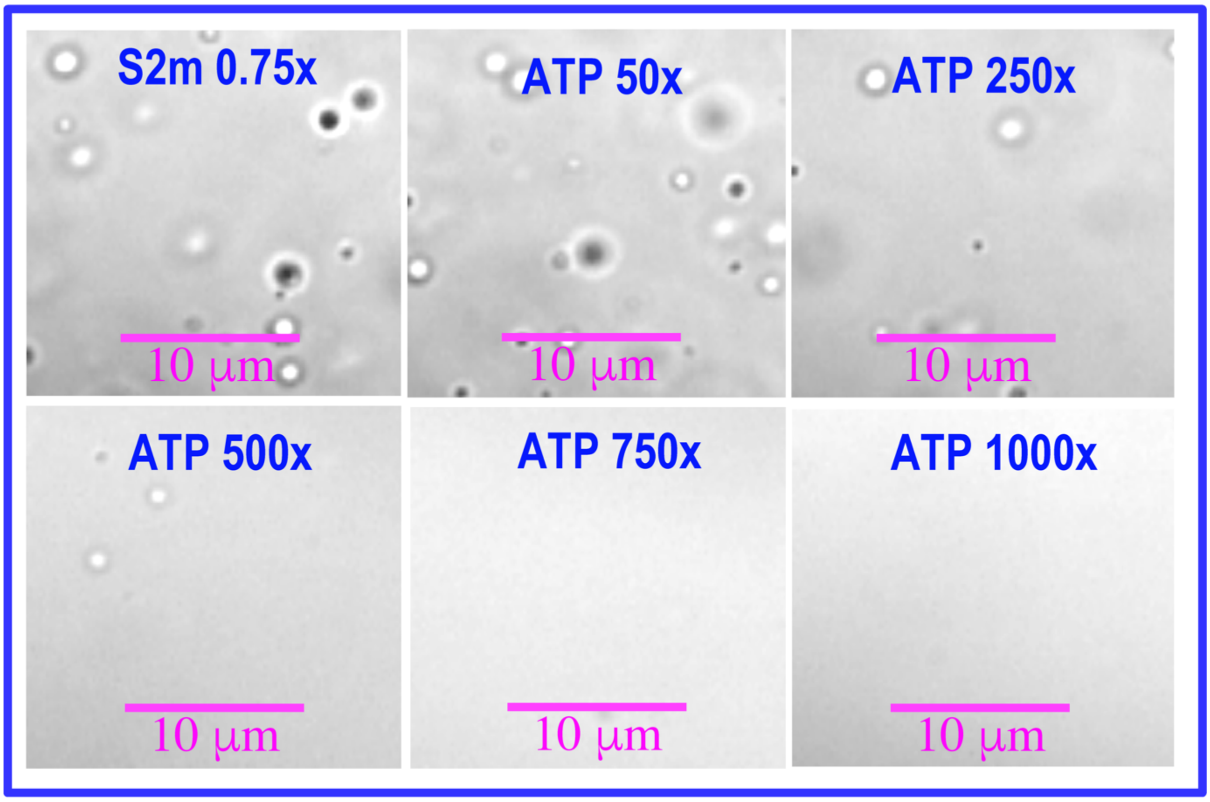

Supplementary Figure 8. ATP dissolves LLPS of the full-length N protein induced by S2m.
DIC images of the full-length N protein in the presence of S2m at 1:0.75 with additional addition of ATP at different ratios.

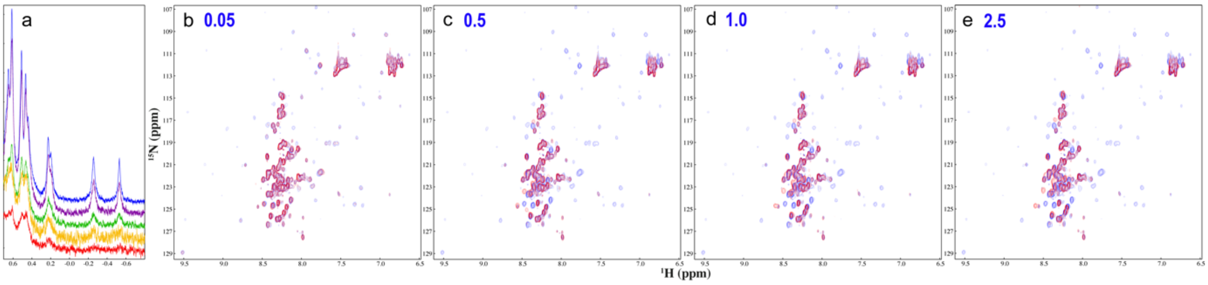

Supplementary Figure 9. NMR view of the binding of S2m with N (247-419).

(a) One dimensional NMR proton spectra of the side-chain regions of N (247-419) in the absence (blue) and in the presence of S2m at 1:0.05 (brown), 1:0.5 (green), 1:1 (yellow) and 1:2.5 (red). Superimposition of HSQC spectra of N (247-419) in the free state (blue) and in the presence of S2m (red) at 1:0.05 (b), 1:0.5 (c), 1:1 (d) and 1:2.5 (e).

Supplementary Table 1. Dissociation constants (Kd) of significantly-perturbed 10 NTD and 4 IDR residues in N (1-249) by ATP-binding

NTD Residue	Kd (mM)	Error (mM)
N48	1.8	0.3
S51	1.9	0.3
T57	1.9	0.3
R89	1.9	0.3
A90	1.9	0.3
R92	1.9	0.2
S105	2.1	0.2
R107	2.1	0.3
A155	2.2	0.4
Y172	2.4	0.5
Average	2.0	
STD	0.2	

IDR Residue	Kd (mM)	Error (mM)
R10	3.0	0.5
R14	2.5	0.3
R32	2.9	0.5
R209	2.7	0.5
Average	2.8	
STD	0.2	
