# Supplementary material for: ATP and nucleic acids competitively modulate LLPS of the SARS-CoV2 nucleocapsid protein
Source: Commun Biol. 2023 Jan 21;6:80. doi: 10.1038/s42003-023-04480-3 (PMC9862227; doi:10.1038/s42003-023-04480-3)
Supplement: Supplementary file 3 — Description of Additional Supplementary Files [file 42003_2023_4480_MOESM3_ESM.docx]

**Description of Additional Supplementary Files**

**File name:** Supplementary Data 1

**Description:** an excel file which contains all turbidity and NMR data used for preparing main figures which include source data for the line graphs in Figs. 2b, 3a, 4b, 5a, 7a, and 7b.
